# A previously healthy 3-year-old female with hypertension, proteinuria, and hypercalciuria

**DOI:** 10.1007/s00467-023-06230-3

**Published:** 2024-01-02

**Authors:** Tao Liu, Wenhong Wang, Zhufeng Liu, Guanghua Pei, Chunxiang Wang, Ying Jiang, Chuyue Pang

**Affiliations:** 1 Department of Nephrology, Tianjin Children’s Hospital (Children’s Hospital, Tianjin University), Tianjin Key Laboratory of Birth Defects for Prevention and Treatment, 238 Longyan Road, Beichen District, Tianjin, 300134 China; 2 Ultrasonography Lab, Tianjin Children’s Hospital (Children’s Hospital, Tianjin University), Tianjin Key Laboratory of Birth Defects for Prevention and Treatment, Tianjin, China; 3 Department of Imaging, Tianjin Children’s Hospital (Children’s Hospital, Tianjin University), Tianjin Key Laboratory of Birth Defects for Prevention and Treatment, Tianjin, China

**Keywords:** Hypertension, Proteinuria, Hypercalciuria

## Abstract

A 3-year-old female patient with no significant medical history presented to her pediatrician with foamy urine. Initial testing revealed moderate proteinuria on qualitative testing, although she was incidentally noted to have severe hypertension (240/200 mmHg). Physical examination of the carotid and femoral areas revealed significant systolic vascular murmurs. Labs showed elevated serum creatinine, hypokalemia, metabolic alkalosis, elevated renin and aldosterone and hypercalciuria. Echocardiography identified ventricular hypertrophy. Computed tomography (CT) of the abdomen and magnetic resonance angiography of the head showed multiple tortuous or interrupted arteries and multiple calcifications in the renal sinus area. B-mode ultrasonography suggested thickening of the carotid and femoral artery walls, with numerous spotted calcifications. Genetic testing revealed that *ABCC6* had a complex heterozygous mutation (exon 24: c.3340C > T and intron 30: c.4404-1G > A). Our panel of experts reviewed the evaluation of this patient with hypertension, proteinuria, hypercalciuria, and vascular abnormalities as well as the diagnosis and appropriate management of a rare disease.

## Case description (Dr. Wenhong Wang, nephrologist, moderator)

The patient was a previously healthy female individual aged 3 years with no significant past medical history who presented to the pediatric clinic in November 2020 with 1 d of increased foaming in urine. Her mother complained that the patient had an upper respiratory tract infection 9 days prior, a runny nose, fever for 4 days, and occasional headaches at ordinary times, but no additional complaints of edema, oliguria, rash, etc. The patient was the first birth of her mother, and no abnormalities were observed during the fetal period. Her mother denied any past or family history of kidney disease. Her father was diagnosed at age 7 with supraventricular tachycardia, followed by intermittent episodes, 1–3 times/year, which could be relieved spontaneously without medication. Her grandfather was diagnosed with supraventricular tachycardia in his 30 s. After that, he averaged about one episode every 2 years. Primary hypertension was found in his 50 s.

On admission, the patient’s vital signs included blood pressure (BP) of 240/200 mmHg (right upper extremity), 230/190 mmHg (left upper extremity), 240/200 mmHg (right lower extremity), 235/195 mmHg (left lower extremity), heart rate of 112 beats/min, normal temperature, and respiratory rate. Her weight was 14.2 kg (25th percentile) and her height was 100 cm (40th percentile). On a physical examination, she was thin. The examination of the head and oropharynx was within normal limits. There was no lymphadenopathy. Lung auscultation revealed no adventitious sounds. The cardiac examination was normal, and no murmurs were detected. Systolic murmurs were observed in the carotid and femoral regions. Her abdomen was soft and non-tender. There were no rashes. Neurological examination revealed no deficits. Initial laboratory studies revealed proteinuria (qualitative 2 +), normal routine blood test results (white blood cells, hemoglobin, and platelets) and C-reactive protein.

Dr. Chuyue Pang and Ying Jiang, as receiving and supervising residents of this patient, what would be the next step in the evaluation and management? Please report back with follow-up lab tests and your own questions.

## Dr. Chuyue Pang and Dr. Ying Jiang (pediatric resident)

In this patient, hypertension was more prominent than proteinuria. A 3-year-old patient with severe hypertension not associated with obesity was considered to have secondary hypertension in the first place. After performing tests (Tables 1 and 2), combined with the child’s medical history and physical examination, we analyzed the etiology of the patient’s hypertension.

**Table 1:** Initial laboratories

Variable	Results on admission	Reference range
Urinary System		
24 h urine		
Urine protein quantitation, mg/24 h	1107	0–150
UCa, mg/kg/24 h	12	0–4
Random urine		
Retinol-binding protein, mg/L	0.73	0–0.70
α1-microglobulin, mg/L	< 6	< 6
β2-microglobulin, mg/L	0.5	0.1–0.3
Microalbumin, mg/L	359.9	0–20.0
Transferrin, mg/L	17.8	< 5
N-acetyl-β-D-glucosidase, U/L	15.5	0.3–12.0
NGAL, ng/mL	7	0–100
Microalbumin/urine creatinine	970	0–30
Plasma		
Urea, mmol/L	5.34	1.78–6.42
Serum creatinine, μmol/L	42	23–37
β2-microglobulin, mg/L	1.38	0.80–2.20
Cystatin C, mg/L	0.7	0.59–1.03
eGFR, ml/(min·1.73 m^2^)	77.8	80.0–120.0
Electrolyte		
Na, mmol/L	136	137–147
K, mmol/L	3.24	3.50–5.30
Cl, mmol/L	91.3	99.0–110.0
Ca, mmol/L	2.32	2.20–2.70
P, mmol/L	1.05	1.10–1.95
Mg, mmol/L	0.82	0.70–0.95
Venous blood gas analysis		
pH	7.569	7.320–7.420
BEblood, mmol/L	9.7	− 3.0–3.0
Blood routine		
WBC count, 10^9^ cells per L	9.23	4.00–10.00
Neutrophils, %	44	45–47
Lymphocytes, %	44	20–40
Inflammatory index		
C-reactive protein, mg/L	0.6	0–5.0
Procalcitonin, ng/L	0.04	0–0.05
Erythrocyto sedimentation rate, mm/h	13	0–20
Pathogenic detection		
Fungus		
1,3-β-D-Glucan, pg/ml	< 10	0–60
Virus	Negative	Negative
IgM of Flu A, Flu B, Parainflu, ADV, and RSV	Negative	Negative
DNA of EBV, HBV and HSV, copies/ml	< 10^3^	< 10^3^
HCVcAg, HCV-Ab	Negative	Negative
Atypical pathogens		
IgM of MP, CPn, COX, LP	Negative	Negative
Bacteria		
Urine culture	Negative	Negative
Antistreptolysin O, IU/ml	7	0–150
Tuberculosis-DNA, copies/ml	< 10^3^	< 10^3^
PPD	Negative	Negative
Endocrine system		
Cortisol, nmol/L	416	133–537(tACQ:6am–10am)
ACTH, pg/mL	5.9	7.2–63.3
17-OHP, ng/mL	< 3.0	< 3.0
PTH, pmol/L	7.05	1.60–6.90
Thyroid function		
TT3, nmol/L	2.90	1.34–2.73
TT4, nmol/L	186.1	78.4–157.4
FT3, pmol/L	8.79	3.69–7.62
FT4, pmol/L	21.56	9.92–19.54
TSH, mIU/L	3.27	0.38–5.33
Hormones		
Follicle-stimulating hormone, IU/L	6.15	0.80–6.22
Luteinizing hormone, IU/L	0.56	0.02–1.84
Prolactin, mIU/L	202.52	63.18–457.92
Estradial, pmol/L	134.0	0–73.4
Testosterone, pmol/L	0.29	0–0.88
RAAS (vertical position)		
Renin, μIU/mL	> 1000	3.11–41.2
Angiotensin I, ng/mL/h	31.58	-
Angiotensin II, pg/mLI	652.8	50.0–120.0
Aldosterone, pg/mL	1748	70–300
Vanilmandelic acid, mg/24 h	5.11	0–13.60
Karyotype	46, XX	46,XX
Adrenal B ultrasound	Normal	-
Thyroid B ultrasound	Multiple calcification lesions	-
Cardiovascular		
Electrocardiograph	High left ventricular voltage	-
Echocardiogram	Left ventricular hypertrophy	-
Creatine kinase, U/L	99	40–200
Creative kinase MB, U/L	1	0–24
Creative kinase MB mass, ng/mL	1.70	0–5.04
Hydroxybutyrate dehydrogenase, U/L	365	72–182
Glutamic oxalacetic transaminase, U/L	34	13–35
Lactate dehydrogenase, U/L	454	120–300
Myohemoglobin, ng/mL	< 21	25–28
Troponin T, ng/mL	< 0.003	0–0.022
Nervous system		
Head MRI	Normal	-
Immunological indicators		
Antinuclear antibody profile	Negative	Negative
Antineutrophil cytoplasmic antibody profile	Negative	Negative
Anticardiolipin antibody	Negative	Negative
Ig A, g/L	1.27	0.2–1.0
Complement 3, g/L	1.04	0.9–1.8
Complement 4, g/L	0.25	0.1–0.4
Metabolic disorders		
Ceruloplasmin, g/L	0.27	0.16–0.45
Blood screening for inherited metabolic diseases, tandem mass spectrometry	Negative	Negative
Urine screening for inherited metabolic diseases, gas chromatography / mass spectrometry	Negative	Negative
Other Indicators		
Serum lipid		
Total cholesterol, mmol/L	5.72	0–5.2
Triglyceride, mmol/L	1.2	0–2.26
High-density lipoprotein, mmol/L	1.48	> 1.86
Low density lipoprotein, mmol/L	3.85	0–2.59
Liver enzymes		
Gamma-glutamyl transpeptidase, U/L	10	7–45
Alanine transaminase, U/L	11	7–40
Total bile acid, μmol/L	22	1–10
Total bilirubin, μmol/L	4.3	0–21
Total protein, g/L	65.1	60–80
Albumin, g/L	37.3	38–54

*Ig* immunoglobulin, *Flu A* influenza A virus, *Flu B* influenza B virus, *Parainflu* parainfluenza virus, *ADV* adenoviridae, *RSV* respiratory syncytial virus, *EBV* EB virus, *HSV* herpes simplex virus, *HBV* hepatitis B virus, *HCV* hepatitis C virus, *MP* Myocoplasima pneumonia, *CPn* Chlamydia pneumoniae, *COX* Coxiella burnetiid, *LP* Legionella pneumophilia, *ACTH* adrenocorticotropic hormone, *17-OHP* 17-hydroxyprogesterone, *PTH* parathyroid hormone, *TT3* serum total triiodothyronic acid, *TT4* total serum thyroxine, *FT3* free triiodothyronine, *FT4* free thyroxine, *TSH* thyroid stimulating hormone, *UCa* urinary calcium quantification, *NGAL* neutrophil gelatinase-associated lipid carrier protein

**Table 2:** Changes in laboratory findings

Variable	D1	D3	D4	D5	D6	D8	D11	D13	D17	Reference range
K, mmol/L	3.24	3.18	-	3.91	-	5.01	-	3.93	3.4	3.5–5.3
Na, mmol/L	136	136	-	133	-	135	-	134	134	137–147
Ca, mmol/L	2.32	2.59	-	2.57	-	2.56	-	2.52	2.33	2.2–2.7
P, mmol/L	1.05	1.37	-	1.57	-	1.39	-	1.09	1.28	1.1–1.95
pH	7.569	7.476	7.480	7.466	7.363	7.421	7.451	7.420	7.519	7.32–7.42
BEb, mmol/L	9.7	6.1	4.4	3.6	0.4	2.4	5.6	3.7	3.3	− 3–3
Urea, mmol/L	5.34	3.2	-	4.3	-	3.74	3.47	2.86	-	1.78–6.42
Scr, μmol/L	42	40	-	36	-	36	24	22	-	23–37
PTH, ng/mL	-	7.05	-	-	-	-	2.63	-	-	1.6–6.9
24hUP, mg/24 h	-	1107	-	-	189.4	-	-	122.1	-	0–150
UCa, mg/kg/24 h	-	12	-	-	9.6	-	-	8.34	-	0–4

*BEb* BEeblood, *Scr* serum creatinine, *PTH* parathyroid hormone, *24hUP* 24-h urinary protein, *UCa* urinary calcium quantification;

-, items not tested

Kidney disorders should be considered first in terms of proteinuria. As the patient was suffering from severe hypertension, we suspected that renovascular hypertension was more likely, such as renal artery stenosis, malformation, local obstruction, or dissection, which are common causes of severe hypertension in children, and scheduled a kidney CT angiography (CTA) for her on day 3 of hospitalization. Unfortunately, we did not find any imaging abnormalities related to bilateral renal arteries on CTA of the abdominal pelvis. However, interestingly, CTA showed unclear splenic artery imaging, reduced splenic perfusion, multiple tortuous and tiny vascular shadows in the tail of the pancreas, uneven and locally delayed perfusion of both renal parenchyma, and multiple calcifications in the renal sinus (Fig. 2). However, the cause of these anomalous findings on the CTA was uncertain. Since hypertension could not be explained by renal artery diseases, we also should identify renal parenchymal diseases. Although she had slightly decreased eGFR and elevated serum creatinine and proteinuria, there was no hematuria, oliguria, edema, or anemia and her measurements were normal, including kidney size and plasma levels of complement, and pathogen tests (Table 1). Therefore, primary glomerulopathy could not explain her high BP. On the other hand, it excludes secondary glomerular diseases, such as systemic lupus erythematosus and hepatitis B-associated glomerulonephritis, in view of normal levels of antinuclear antibody spectrum, antiphospholipid, and hepatitis B antibodies. Her family history of kidney disease was ruled out, and examination of her funduscopy, brainstem auditory evoked potential, and her parents’ urine routine were all normal; consequently, it was irrespective of hereditary kidney diseases. Her renin angiotensin aldosterone (RAA) levels were significantly elevated, which did not support renal tubular diseases, such as Liddle syndrome. In addition, care should be taken to identify other secondary factors for hypertension, such as endocrine disorders, cardiovascular diseases, neurological disorders, intoxication or sleep apnea, and acute intermittent porphyria, all of which could have been ruled out based on her history, physical examination, and additional tests (Tables 1 and 2).

**Fig. 1:**
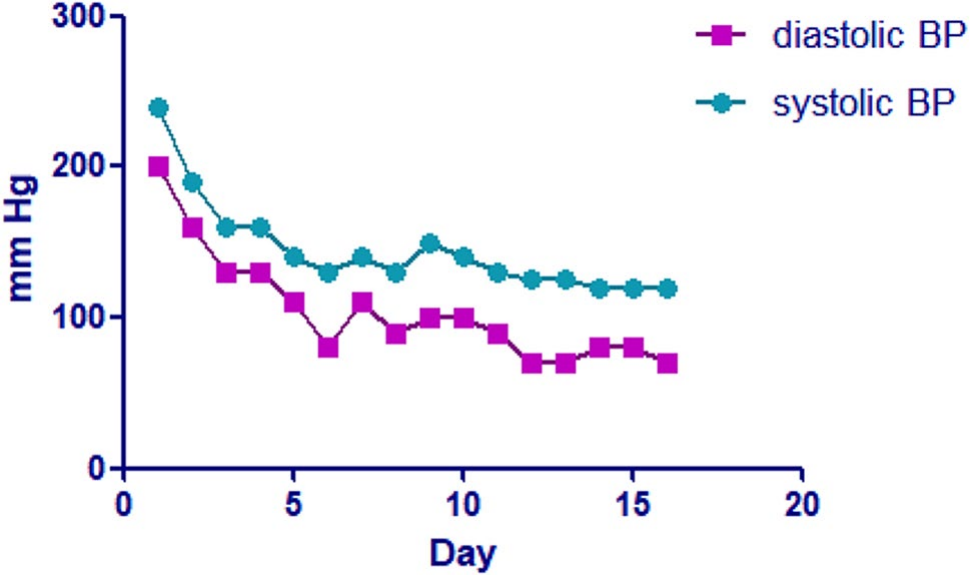
BP changes during hospitalization. Nifedipine 5 mg per 6 h was given on day 1 and betaloc 12.5 mg per 12 h on day 3

Given her high BP, the next management step was to focus on smooth BP reduction. There were no signs of acute severe hypertension that we commonly see, such as significant headaches, dizziness, blurred vision, and vomiting. As a result, it was assumed that the patient’s hypertension could also be a chronic process and that the patient was already tolerating it. Therefore, we chose to first try oral rather than intravenous treatment. It was not known if she had bilateral renal artery stenosis prior to the abdominal pelvic CTA exam, so we did not initially select ACEI/ARB. Because the patient had severe hypertension, a calcium channel blocker (nifedipine 5 mg q6h) was chosen. In the first day her BP went from 240/200 to 190/160 mmHg. In consideration of her increased heart rate, we later administered her combined oral treatment with β receptor blocker-metoprolol tartrate (12.5 mg per 12 h). We gradually replaced nifedipine with the long-acting amlodipine 5 mg qd on day 7 and her BP decreased steadily to 130/70 mmHg (Fig. 1).

So far, the difficulty was that we did not find the cause of hypertension from various systemic diseases. In addition, hypercalciuria was observed during the examination. Could one pathology account for hypertension, proteinuria, hypercalciuria, or vascular abnormalities in the pancreas and spleen?

## Dr. Wenhong Wang

**Fig. 2:**
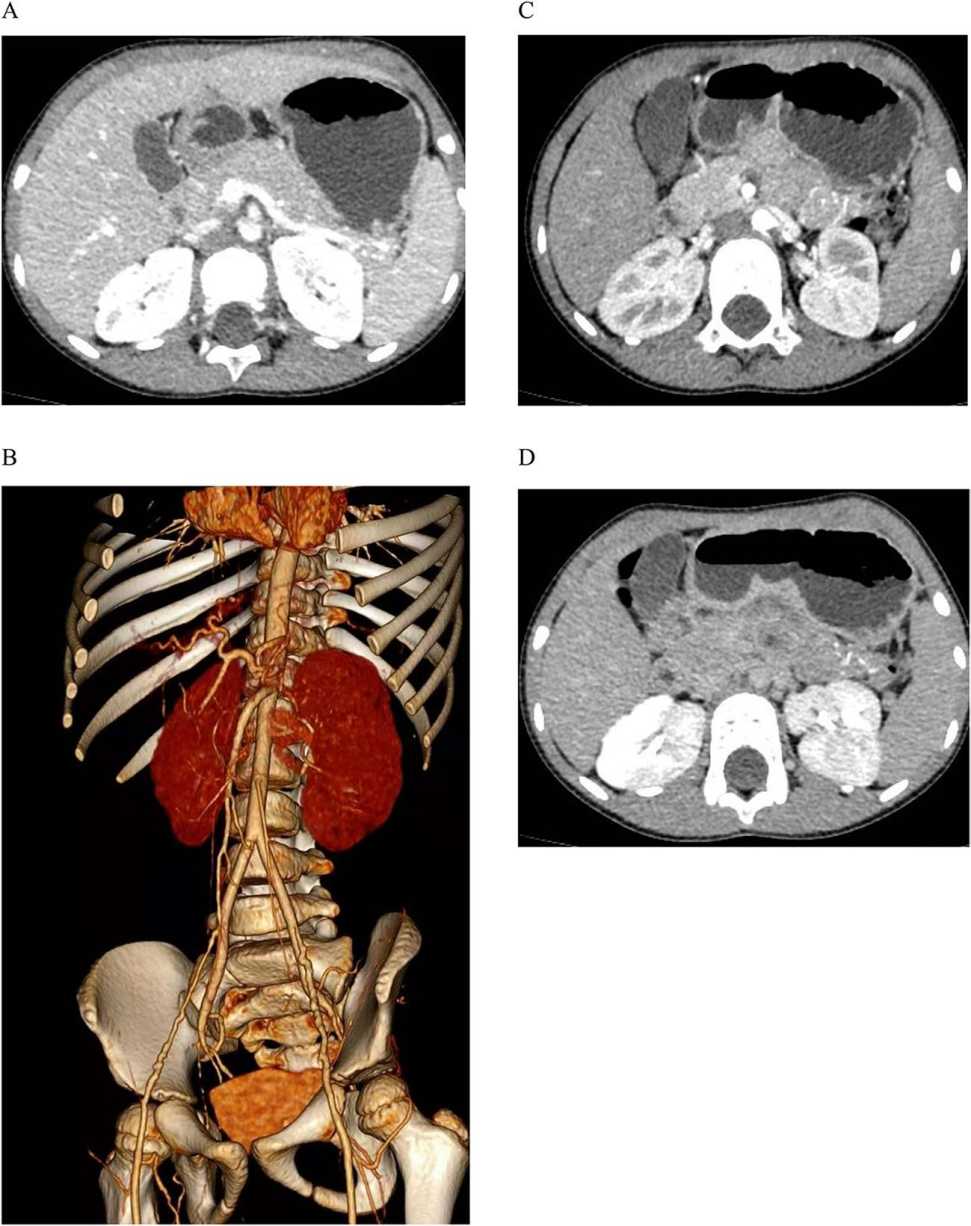
CTA of the abdominal pelvis.** A** Splenic artery imaging was not clear and splenic blood perfusion was reduced. **B** There were multiple tortuous and tiny vascular shadows in the tail of the pancreas. **C** No stenosis or malformation of the renal arteries was found. **D** Perfusion of the renal parenchyma is not uniform

We summarized the patient’s clinical characteristics. She was 3 years old with severe hypertension, kidney injury (proteinuria, elevation of serum creatinine, and slightly decreased eGFR), significantly increased RAA levels, abnormal abdominal pelvic CTA findings (blurred and tortuous arteries of the spleen and pancreas), renal sinus calcification, and hypercalciuria (Fig. 2).

Dr. Tao Liu, as a nephrology resident, what train of thought should we analyze next and what other tests should we perform?

## Dr. Tao Liu (nephrology resident)

**Fig. 3:**
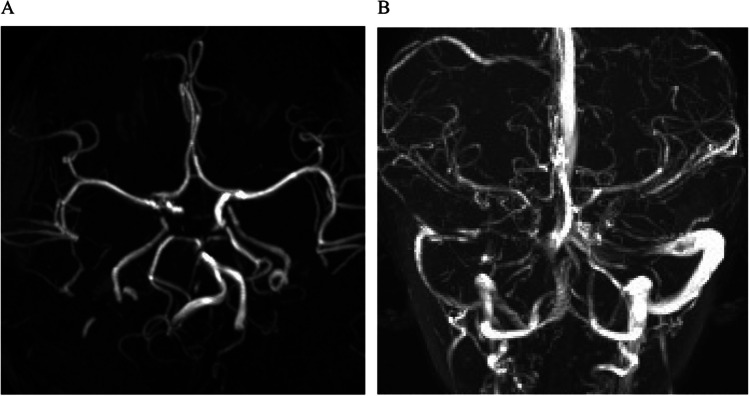
Head MRA and MRV. **A** MRA displayed that bilateral internal carotid arteries are slender and locally interrupted, multiple lumen development was interrupted in the basilar artery and bilateral posterior cerebral artery proximal and bilateral posterior communicating artery-posterior cerebral artery junction, and the lumen of the right anterior cerebral artery was narrow at the origin. **B** MRV displayed that the right sigmoid sinus and internal jugular vein were tenuous

By comparison, it was found that her elevated RAA levels were more significant, so I think we can further analyze her etiology from the perspective of high-renin hypertension which includes the following categories of diseases such as pheochromocytomas and paragangliomas (PPGLs) [1], renin-secreting tumors [2], renal vascular hypertension [3], and hypertension with renal microvascular disease [4]. Given the normal blood and urine catecholamine secretion levels and 24-h urine vanilmandelic acid level (Tables 1 and 3), PPGLs were not considered. Renin-secretory tumor could not explain her vascular abnormalities in the spleen or pancreas, and there was no identified mass on abdominal pelvic CTA to support a renin-secreting tumor. After excluding the diagnosis, only hypertension with renal microvascular disease was left as the disease category for further consideration. Given the vascular abnormalities in the spleen and pancreas, it was thought that her uneven and locally delayed perfusion may also be a sign of microvascular disease in kidneys or might only be a partial indication of systemic vasculopathy. To prove that she had systemic vasculopathy, we further refined her head magnetic resonance angiography (MRA) and magnetic resonance venography (MRV) in light of her headache and found that the bilateral internal carotid arteries were slender and locally interrupted. Multiple lumen development was interrupted in the basilar artery and bilateral posterior cerebral artery proximal and bilateral posterior communicating artery-posterior cerebral artery junction; the lumen of the right anterior cerebral artery was narrow at the origin, and the right sigmoid sinus and internal jugular vein were tenuous (Fig. 3). Thus, her head MRA and MRV findings further confirmed our judgment that she had systemic vasculopathy.

**Table 3:** Catecholamine secretion levels

Variable	Results on admission	Reference range
Plasma		
NMN, pg/mL	122.0	< 145
MN, pg/mL	32.2	< 62
NMN + MN, pg/mL	154.2	< 207
24 h urine		
DA, µg/24 h	91.6	0–600
NE, µg/24 h	13.5	0–90
E, µg/24 h	0.9	0–20
NMN, µg/24 h	68.7	52–247
MN, µg/24 h	22.5	42–135
NMN + MN, µg/24 h	91.2	94–382

*NMN* free normetanephrine, *MN* free metanephrine, *DA* free dopamine, *NE* free norepinephrine, *E* free epinephrine

## Dr. Wenhong Wang

So what are the main diseases that affect children with systemic vasculopathy? What is the relationship between systemic vascular diseases and hypercalciuria? Dr. Zhufeng Liu, as deputy chief physician of pediatric medicine, would you like to express your opinion?

## Dr. Zhufeng Liu (deputy chief physician, pediatric nephrology department)

Severe hypertension with systemic vasculopathy in children is common in multiple arteritis and fibromuscular dysplasia (FMD) [5]. Although the abdominal pelvic CTA showed multiple abnormal blood vessels, multiple arteritis was not considered because the patient had no dizziness, palpitations, or unexplained fever, BP difference between the upper and lower extremities was less than 20 mmHg, inflammatory markers such as CRP and ESR were normal, and echocardiography showed no aortic involvement other than left ventricular hypertrophy. In combination with the presence of multiple, interrupted, and slender arteries, FMD should be considered. Classical CTA findings include the “string-of-beads” appearance of medial FMD [6], which this patient did not exhibit on the abdominal pelvic CTA. FMD also failed to explain the hypercalciuria and multiple calcifications in the renal sinus. Therefore, FMD was ruled out in this case. Because she had severe hypertension with systemic vasculopathy and hypercalciuria, we thought of a rare disease called generalized arterial calcification in infants (GACI) [7]. Consequently, we need to search for more evidence of arterial calcification in the next steps.

## Dr. Wenhong Wang

In view of the need to determine the basis of arterial calcification in GACI, we invited Chunxiang Wang, Radiology Department Director, and Guanghua Pei, Ultrasound Department Director, to analyze the vascular imaging manifestations of this patient.

## Dr. Chunxiang Wang (radiology department director) and Dr. Guanghua Pei (chief physician, ultrasound department)

**Fig. 4:**
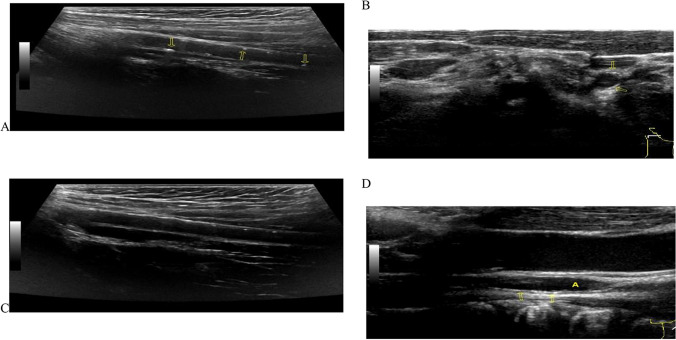
B-ultrasound images of the carotid and femoral arteries. The wall of the carotid and femoral arteries were thickened with multiple mottled calcifications. **A** Right femoral artery; **B** right carotid artery; **C** Left femoral artery; **D** Left carotid artery

Upon admission, the patient underwent abdominal and pelvic CT and CTA, head MRA and MRV in the imaging department. The most commonly calcified arteries in late-onset GACI are the thoracic and abdominal arteries such as the coronary, renal, pulmonary, and aorta arteries [8]. Unfortunately, we did not find evidence of arterial calcification in the abdomen, pelvis, or intracranial vessels. We later performed a chest CT on the patient but again found no arterial calcification. Given the large amount of radiation that patients receive in the short term, we can perhaps continue to search for the basis of vascular calcification through other means such as vascular ultrasound. An ultrasound examination of the blood vessels found that the walls of the carotid and femoral arteries were thickened with multiple mottled calcifications (Fig. 4). Therefore, these findings should be used as diagnostic tools for GACI. As to why CT did not detect arterial calcification but ultrasound did, we considered that it might be related to the following factor: the patient was a late case with an atypical onset. Arterial calcification may not begin in the chest and abdominal arteries, which are common sites of calcification, but rather in the limb and carotid arteries. While CT is the imaging modality of choice for the evaluation of calcifications [7], ultrasound allows for noninvasive diagnosis, assessment of severity, and monitoring of progression [9].

Although CTA did not reveal renal artery stenosis, the possibility of homogeneous thickening of the renal artery intima could not be ruled out nor the possibility of smaller branch renal artery stenosis. We suggested advanced renal angiography, but the family declined our advice. Additionally, B-scan ultrasonography (B-scan) of the kidneys was performed and both kidneys were normal in size, but the parenchymal echo was enhanced, and multiple strong echo spots which were thought to be crystals were found in the calyces. These may be manifestations of GACI on B-scan of the kidneys.

## Dr. Zhufeng Liu

We summarized the clinical characteristics of the patient. She was 3 years old with severe hypertension, kidney injury, significantly increased RAA levels, hypercalciuria, multiple systemic vascular abnormalities, calcification of the carotid and femoral arteries, and calcification of the renal sinus area. In summary, we suspected that the patient had a GACI. After obtaining informed consent from her parents, we performed genetic sequencing of her entire exon and validated the genes of her parents. The results showed that *ABCC6* had a complex heterozygous mutation, exon 24: c.3340C > T and intron 30: c.4404-1G > A. Her mother carried c.3340C > T and her father carried c.4404-1G > A. According to the guidelines of the American Society of Medical Genetics and Genomics, both mutations were determined to be pathogenic variants. The patient was eventually diagnosed with GACI.

## Summary (Dr. Wenhong Wang)

Here, we focus on the differential diagnosis of hypertension in early childhood, while reporting the diagnosis, evaluation, and treatment of a 3-year-old Asian patient with GACI which is a rare genetic disease that affects the circulatory system in general, and the large and medium-sized arteries in particular, throughout the body. We summarize the following aspects of this case next.

### Gene mutations and pathogenesis of GACI

GACI is a rare autosomal recessive genetic disorder caused mostly by mutations in the *ENPP1* or *ABCC6* genes, which cause GACI type 1 and GACI type 2, respectively. The number of affected individuals with *ABCC6* variants was small versus affected individuals with *ENPP1* variants (9% versus 67%) [7, 10]. Our case had GACI type 2, due to *ABCC6* compound heterozygous mutations. So far, only a dozen *ABCC6* gene mutations associated with GACI have been reported [11, 12], including missense mutation c.3340C > T, while c.4404-1G > A is a novel mutation site. *ABCC6* is responsible for coding the protein of ATP-binding cassette subfamily C, member 6 (ABCC6), whose function is not yet clear [13]. It is assumed that *ABCC6* is a transmembrane transporter expressed primarily in hepatocytes and proximal tubular epithelial cells of the kidney [14, 15]. *ABCC6* has been shown to influence extracellular ATP levels and therefore plays a role in the pyrophosphate (PPi) generating pathway [15, 16]. PPi inhibits the crystallization and the growth of calcium phosphate crystalline phases such as hydroxyapatite. Patients with GACI as well as Abcc6^−/−^ mice have a reduced plasma PPi level which promotes the deposition of hydroxyapatite in arteries and organs [17].

### Clinical features of GACI and the new manifestations of this case

GACI is characterized by infantile onset of widespread arterial calcification and/or narrowing of large and medium-sized vessels, resulting in cardiovascular findings (including heart failure, respiratory distress, edema, cyanosis, hypertension, and/or cardiomegaly) [8, 18–20]. Additional findings can include typical pseudoxanthoma elasticum (PXE)-like skin and retinal manifestations, periarticular calcifications, nephrocalcinosis, development of rickets after infancy, cervical spine fusion, and hearing loss [7, 19]. There is a similar prevalence of GACI phenotypes between *ENPP1* and *ABCC6* mutations, including arterial calcification (77.2% and 89.5%, respectively), organ calcification (65.8% and 84.2%, respectively), and cardiovascular complications (58.4% and 78.9%, respectively) [21]. But in contrast to the *ABCC6* mutations, the incidence of *ENPP1* mutations was higher in terms of mortality, rickets, joint calcification, hearing complications, and neurological complications [21].

The majority of GACI patients had an onset during fetal life or within 1 year of birth, and the median age of onset was 3 months [6]. The case in this paper had a late onset age of 45 months. Late-onset (≥ birth 1 week) GACI most commonly involved the coronary, renal, and pulmonary arteries [6]. However, in our patient, arterial calcification occurs first in the lower extremities and in the cervical arteries, which may be related to different genetic phenotypes. During follow-up, she gradually developed scattered calcifications in the aorta abdominalis, superior mesenteric artery, arteria mesenterica inferior, bilateral iliac artery, bilateral upper limb arteries, bilateral subclavian arteries, bilateral lower limb arteries, aortic valve, mitral valve, pulmonary valve, left ventricular endocardium, and the circumflex branch of the coronary artery as measured by vascular B-scan, echocardiography, and coronary artery CTA examination (Table 4). Her left ventricular hypertrophy gradually eased (Table 5) which may be related to smooth blood pressure control. Arterial stenosis, which has been reported, does not necessarily occur at the same time as arterial calcification, and can occur over time in the same vessels as calcification [22]. Our patient also had no evidence of arterial stenosis at the time of diagnosis. However, at the fourth month after the diagnosis of GACI, coronary CTA reexamination revealed the stenosis of the anterior descending coronary artery, circumflex branch and right coronary artery, and the stenosis degree was less than 50% (Table 4). At the same time, the vascular B-scan also found that the flow rate and resistance of both renal arteries increased (Table 5) which further confirmed the possibility of renal microvascular stenosis. In addition, the middle and distal lumen of her right profunda femoral artery were occluded. The cardiovascular complications of GACI (*ABCC6* mutation) are mostly reported as heart failure, hypertension, and respiratory failure, while severe hypertension like our case has only been reported in rare cases. We speculate that BP may not be severely elevated in patients with concurrent heart failure. In addition, in terms of organ calcification, we identified some previously unreported abnormalities, including thyroid calcification with abnormal thyroid function and kidney crystallization with hypercalciuria, but found no joint calcification commonly reported in the literature [7]. At the beginning of the disease, B-scan revealed multiple calcifications of the thyroid, accompanied by abnormal thyroid function, showing elevated FT3 and FT4 levels but normal TSH levels, and after a short period of BP control, her thyroid function improved spontaneously. As a result, thyroid dysfunction may be a secondary alteration of GACI for hypertension or organ calcification.

**Table 4:** Manifestations of coronary CTA

	Number of calcified plaques	Number of non-calcified plaques	Degree of stenosis		
			< 50%	50–70%	> 70%
2021.12.30	0	3 (LAD2, RCA1, RCA2)	LAD2	-	-
2022.2.25	1 (CX)	6 (LAD1,LAD2, CX1, RCA1, RCA2, RCA3)	LAD1,LAD2, CX1, CX2, RCA1, RCA2, RCA3	-	-
2022.11.03	0	8 (LM, LAD1,LAD2, CX1, CX2, RCA1, RCA2, RCA3)	LAD1,LAD2, LAD3, CX1, CX2, CX3, RCA1, RCA2, RCA3, AM	-	-

*LAD* left anterior descending branch, *LM* left main coronary artery, *RCA* right coronary artery, *CX* circumflex branch, *AM* right marginal branch

**Table 5:** Manifestations of echocardiography and renal artery B-scan

	2021.11.27	2021.12.28	2022.2.24	2022.5.20	Reference range
Echocardiography					
Interventricular septal depth (mm)	7	6	5	4	5.1 ± 0.7
Left ventricular posterior wall depth (mm) Calcification	9 no	7 Aortic valve, Mitral valve	6 Aortic valve, Mitral valve, Pulmonary valve, Left ventricle endocardial	4 Aortic valve, Mitral valve, Pulmonary valve	5.1 ± 0.7 no
Renal artery PSV (cm/s)					
Right renal artery initiation segment	-	54	151	-	53.8 ± 3.2
Left renal artery initiation segment	-	60	128	-	54.2 ± 4.0
Right interlobar renal artery	-	26	19	-	-
Left interlobar renal artery	-	19	18	-	-
Initial internal diameter of renal artery (mm)					
Right renal artery	-	2.5	2.4	-	2.9 ± 0.27
Left renal artery	-	2.4	2.4	-	3.03 ± 0.49

*PSV* peak systolic velocity

### GACI and kidney disease

To date, which specific area of the kidney is affected by GACI has been scarcely reported. To our knowledge, in patients with GACI, kidney disease mainly presents as nephrocalcinosis, renovascular hypertension, and kidney failure. Acute kidney failure commonly occurs in infancy [8, 23], while in adulthood it is indicated by a decline in renal tubular reabsorption function [19]. In our case, there was a slightly decreased eGFR and an increase in serum creatinine and proteinuria, which returned to the normal range after antihypertensive therapy. Therefore, we consider that these abnormalities may be related to hypertension and hypoperfusion caused by minor renal vessel stenosis, which is a chronic progressive process. Despite the fact that severe forms of chronic kidney diseases (CKD) have not been described as a consequence of GACI, we still cannot ignore the risk of CKD in adulthood of GACI. Second, the kidney is one of the most common sites of organ calcification in GACI. In addition to renal artery calcification, renal calcification can also manifest as nephrocalcinosis [11]. While medullary nephrocalcinosis is likely a complication from treatment of rickets, cortical nephrocalcinosis likely represents a consequence of ischemia [7]. When *Abcc6*^−/−^ and *Enpp1*^−/−^ mice (two established mouse models of ectopic mineralization) were placed on the acceleration diet, they revealed extensive mineralization in the kidney interstitium, primarily affecting the medullary tubules as well as arcuate and renal arteries, whereas when on standard rodent diet, developed nephrocalcinosis only at very late age [24], which may explain why nephrocalcinosis rarely occurs in infants. Of note, in our case, we did not find nephrocalcinosis but did find multiple crystals in the bilateral calyces, which has not been reported in the previous literature and is indeed considered to be one of the common features of another *ABCC6* mutant disease, pseudoxanthoma elasticum (PXE) [25]. In addition, this patient had hypercalciuria, which had also not been described previously. Mutations in *ENPP1* or *ABCC6* lead to the deposition of excess hydroxyapatite particles in the Henle basement membrane of the renal tubules. These particles then grow extending into the medullary interstitium and further towards the papillary surface. The mineral syncytium can lie beneath the uroepithelium and may become exposed to the urinary environment after the loss of uroepithelial integrity [24]. Hypercalciuria could therefore be involved first in the deposition of excess hydroxyapatite particles [24]. The patient was treated with bisphosphonates at a later period and the urinary calcium levels returned to normal for a time, but there was no significant change in the crystallization of the calyces. Regarding the proteinuria of the patient, we considered the following factors to be relevant: her proteinuria gradually normalized as her BP decreased, and it was assumed that hypertension was also involved in the production of proteinuria; CTA showed uneven and delayed local perfusion of the renal parenchyma, and increased renal arterial resistance was found during follow-up, thought to be associated with stenosis of minor arteries in the kidneys, causing ischemic nephropathy. 

### The differential diagnosis of GACI (ABCC6 mutations)

*ABCC6* mutations can manifest as two different diseases, GACI and PXE, which overlap in phenotype and need to be distinguished. Classic PXE, caused by *ABCC6* variants, appears as soon as the second decade of life with skin changes and is characterized by mineralization and fragmentation of elastic fibers in the skin, eyes, and cardiovascular system. In contrast, GACI has a younger onset age, a higher mortality rate, an earlier onset of arterial calcification, and a lower incidence of skin and eye lesions. The most commonly calcified arteries in GACI are thoracic and abdominal arteries such as coronary, renal, pulmonary, and aortic arteries. Whereas, PXE has a moderate onset and most affected individuals live a normal life span. Skin and eye lesions occur mostly in the first or second decade of life. Vascular signs (except for claudication) usually become apparent years after the onset of skin and ocular changes. The primary clinical expression of the arterial wall mineralization is intermittent claudication in both lower and upper limbs and peripheral artery disease [26]. In our case, the patient was diagnosed with GACI due to her early age, arterial calcification, and lack of skin and eye lesions. She may, of course, develop PXE-like skin and eye lesions over time, which we should continue to watch.

### Treatment and follow-up for GACI

Up to now, there is no curative treatment for GACI. The goal of early diagnosis and treatment in children with GACI is to promote the regression of arterial calcifications and cessation of the development of arterial stenoses. Arterial calcifications may decrease or disappear spontaneously, after treatment, but arterial stenoses usually persist that can cause significant clinical ischemic complications [22]. Classic drugs that promote the regression of arterial calcification are bisphosphonates such as etidronate, pamidronate, and risedronate. This patient had no calcifications in the thoracic and abdominal arteries and no evidence of calcification on a coronary artery CTA performed 1 month after her GACI diagnosis; therefore, no bisphosphonate therapy was initiated immediately. However, 3 months after the diagnosis of GACI, coronary artery calcification was detected during follow-up monitoring, and intravenous pamidronate was initiated. The first day was 0.25 mg/kg, and second and third days were 0.5 mg/kg. After that, treatment was every 2 months as a course of treatment (0.5 mg/kg /d × 3 days) for 4 consecutive courses. Repeated coronary artery CTA, performed 1 year after diagnosis, and compared to the previous coronary artery CTA, reported less coronary calcification. However, bisphosphonates improve calcium salt deposition but do not delay the progression of arterial stenosis, as also shown in our case (Table 4), which was treated with aspirin at the cardiologist’s recommendation. During the bisphosphonate administration, the patient did not develop rickets and only showed a transient decrease in blood calcium, which quickly returned to normal upon administration of calcium supplements. In addition, arterial hypertension is also prominent in GACI. Since hypertension in GACI is most likely caused by stenosis of the renal arteries, it may be beneficial to use ACEI or ARB. Moreover, ectopic arterial calcification can manifest in any systemic organ, thus requiring individualized treatment for each organ dysfunction such as cardiac failure, rickets, PXE-like eye lesions, and hearing loss. These systemic diseases need to be tracked by a comprehensive medical team involving multiple specialties for their management.

## Conclusions

Secondary hypertension is the most severe form of hypertension in children. In addition to diseases of various systems, rare diseases should be considered. We hope to provide a reference for general practitioners by introducing a differential diagnosis for hypertension in this patient. In addition, GACI is a rare disorder in children, and the possibility of GACI should be considered in patients with atypical onset conditions such as hypertension, proteinuria, and hypercalciuria. B-scan, CT, and genetic testing are helpful for early detection and diagnosis of GACI.

## Data Availability

The authors confirm that the data supporting the findings of this study are available within the article [and/or its supplementary materials].

## Abbreviations

ABCC6: ATP-binding cassette subfamily C, member 6
ACTH: Adrenocorticotropic hormone
BP: Blood pressure
B-scan: B-scan ultrasonography
CAG: Angiogram
CAH: Congenital adrenal cortex hyperplasia
CTA: Computed tomography angiography
eGFR: Estimated glomerular filtration rate
FMD: Fibromuscular dysplasia
GACI: Generalized arterial calcification in infants
MRA: Magnetic resonance angiography
MRV: Magnetic resonance venography
PPGLs: Pheochromocytomas and paragangliomas
PXE: Pseudoxanthoma elasticum
RAA: Renin-angiotensin-aldosterone
